# The Paradox of Modern Technology in Standardizing Thermal Liver Ablation: Fostering Uniformity or Diversity?

**DOI:** 10.1007/s00270-024-03846-2

**Published:** 2024-09-03

**Authors:** Coosje A. M. Verhagen, Ariadne L. van der Velden, Reto Bale, Elena Bozzi, Laura Crocetti, Alban Denys, Gonnie C. M. van Erp, Faeze Gholamiankhah, Giorgio Greco, Pim Hendriks, Robrecht R. M. M. Knapen, Hicham Kobeiter, Rodolfo Lanocita, Martijn R. Meijerink, Franco Orsi, Alice Phillips, Hossein Rahmani, Maarten L. J. Smits, Marco J. L. van Strijen, Ronald M. van Dam, Christiaan van der Leij, Mark C. Burgmans

**Affiliations:** 1 Department of Radiology, Leiden University Medical Center, Leiden, The Netherlands; 2 Department of Radiology, Maastricht University Medical Center, Maastricht, The Netherlands; 3 Department of Radiology, Medical University Innsbruck, Innsbruck, Austria; 4 Department of Radiology, Azienda Ospedaliero Universitaria Pisana, Pisa, Italy; 5 Department of Radiology and Interventional Radiology, CHUV University of Lausanne, Rue du Bugnon, Lausanne, Switzerland; 6 Department of Radiology, Foundation IRCCS Istituto Nazionale Tumori, Milan, Italy; 7 Radiology Department, H. Mondor Hospital, Assistance Publique-Hôpitaux de Paris, University Paris Est Creteil, Creteil, France; 8 Department of Radiology, Amsterdam University Medical Center, Amsterdam, The Netherlands; 9 Department of Interventional Radiology, European Institute of Oncology, IRCCS, Milan, Italy; 10 Department of Radiology, IRCCS Foundation Ca’ Granda Ospedale Maggiore Policlinico, Milan, Italy; 11 Department of Radiology and Nuclear Medicine, University Medical Center Utrecht, Utrecht, The Netherlands; 12 Department of Radiology, St. Antonius Hospital, Nieuwegein, The Netherlands; 13 Department of Surgery, Division of Hepato-Pancreato-Biliary and Oncology, European Surgery Center Aachen Maastricht, Maastricht UMC+, Maastricht, The Netherlands

**Keywords:** Thermal ablation, Liver tumor, Interventional oncology, Modern technology, Standardization

## Abstract

**Purpose:**

Currently, significant medical practice variation exists in thermal ablation (TA) of malignant liver tumors with associated differences in outcomes. The IMaging and Advanced Guidance for workflow optimization in Interventional Oncology (IMAGIO) consortium aims to integrate interventional oncology into the standard clinical pathway for cancer treatment in Europe by 2030, by development of a standardized low-complex-high-precision workflow for TA of malignant liver tumors. This study was conducted at the start of the IMAGIO project with the aim to explore the current state and future role of modern technology in TA of malignant liver tumors.

**Materials and Methods:**

A cross-sectional questionnaire was conducted followed by an expert focus group discussion with core members and collaborating partners of the consortium.

**Results:**

Of the 13 participants, 10 respondents filled in the questionnaire. During the focus group discussion, there was consensus on the need for international standardization in TA and several aspects of the procedure, such as planning based on cross-sectional images, the adoption of different techniques for needle placement and the importance of needle position- and post-ablative margin confirmation scans. Yet, also considerable heterogeneity was reported in the adoption of modern technology, particularly in navigational systems and computer-assisted margin assessment.

**Conclusion:**

This study mirrored the current diversity in workflow of thermal liver ablation. To obtain comparable outcomes worldwide, standardization is needed. While advancements in tools and software hold the potential to homogenize outcome measurement and minimize operator-dependent variability, the rapid increase in availability also contributes to enhanced workflow variation.

**Graphical Abstract:**

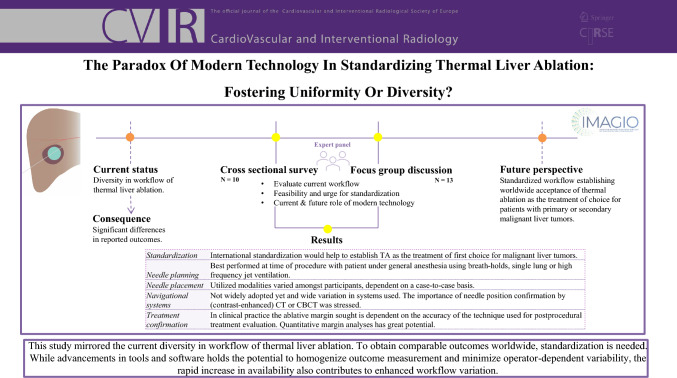

**Supplementary Information:**

The online version contains supplementary material available at 10.1007/s00270-024-03846-2.

## Introduction

Thermal ablation (TA) is an effective treatment for malignant liver tumors with clinical outcomes equivalent to surgical resection [1–5]. Yet, reported outcomes differ significantly between centers and countries, due to variability in patient selection, ablation technique and treatment evaluation [3, 6–8]. This hampers worldwide acceptance of ablation as treatment of first choice.

A multitude of developments in tools and software has become available [8–10]. The integration of these evolvements holds the potential to optimize technical success and clinical outcomes. However, the use of these techniques is still limited, mainly due to the imbalance of the additional costs and procedural benefits. Furthermore, various logistic and technical hurdles along with a certain conservatism among potential users hamper implementation in clinical practice.

An Innovative Health Initiative grant (No. 101112053) was rewarded to the ‘IMaging and Advanced Guidance for workflow optimization in Interventional Oncology’ (IMAGIO) consortium, consisting of 30 partners in academia, healthcare institutions and industry [11]. The purpose of the IMAGIO consortium is to make interventional oncology part of the standard clinical care pathway to cancer treatment in Europe by 2030. Among the main deliverables are to develop a standardized, accessible, and low-complexity-high-precision workflow for liver tumor TA *and* artificial intelligence algorithms to support patient selection, treatment planning, needle guidance and treatment evaluation.

At the start of the IMAGIO project, an international expert focus group discussion (FGD) was organized to evaluate the current and future role of modern technology in TA.

## Materials and Methods

This was a mixed-method study comprising a semi-structured FGD preceded by a cross-sectional questionnaire.

1. Cross-sectional questionnaire:

Three weeks prior to the FGD, an online questionnaire was conducted comprising 15 questions, each accompanied by a rationale. The questions were designed by the FGD moderators (MB, CvL), also principal investigators in the IMAGIO project, and pertained to deliverables of the IMAGIO project (appendix A, Table 1).

**Table 1:** Background of participants and respondents

	Participants, n (%)	Respondents, n (%)
Total	13 (100%)	10 (100%)
Country		
Austria	1 (7.7%)	1 (10%)
France	1 (7.7%)	1 (10%)
Italy	4 (30.8%)	2 (20%)
Netherlands	6 (46.2%)	5 (50%)
Switzerland	1 (7.7%)	1 (10%)
Medical specialty		
Interventional radiologist	12 (92.3%)	10 (100%)
Hepatobiliary surgeon	1 (7.7%)	0 (0%)

2. Focus group discussion:

The FGD took place during the Cardiovascular and Interventional Radiological Society Europe (CIRSE) 2023 congress in Copenhagen. The results of the questionnaire were anonymously presented. The FGD was semi-structured with open-ended predefined questions, facilitated by two experienced moderators.

Two independent researchers (CV and PH) documented the raw data during the session, transcribed it and established inter-coder agreement and thematic analysis, followed by two peer debriefing sessions.

### Participants

Since this study is part of the EU-funded IMAGIO study, the target audience consisted of European experts in the field. The selected body consisted of core members (n = 7) and collaborative partners (n = 6) of the IMAGIO consortium (Table 1). Twelve participants were employed by academic centers. One participant practiced in a large teaching hospital.

## Questionnaire and Focus Group Discussion Findings

Appendix A, Table 1 illustrates the quantitative results of the questionnaire. Per topic, the results of the questionnaire and FGD are summarized.

### Standardization

Nine respondents emphasized the importance of international standardization of liver tumor ablation, while one respondent did not due to the unclear specification of the proposed standardization. During the FGD, there was consensus that standardization would help to establish TA as treatment of first choice for malignant liver lesions. To compete with surgery, TA should universally be excellent with limited operator-dependency. Participants noted that some standardization already exists through guidelines from CIRSE, the Society of Interventional Radiology and the Society of Interventional Oncology.

### Software-assisted Preprocedural Planning

The FGD revealed that all participants currently use preprocedural cross-sectional computed tomography (CT) and/or magnetic resonance imaging images for patient selection and needle trajectory planning. There was consensus that needle planning is best performed periprocedural, preferably with the patient under general anesthesia using respiratory control (i.e., breath-holds, single-lung or high-frequency jet ventilation). It was stressed that the position and morphology of the liver depend on the patient’s position and breathing and thus may vary considerably. Software-assisted planning *prior* to the intervention using pre-procedural images was therefore considered of limited use. *Pre-procedural* planning software*,* however, could be beneficial in hands-on simulation training for trainees and inexperienced interventional radiologists.

### Needle Placement

Four of the respondents considered ultrasonography (US) as the modality of first choice as it allows real-time needle placement. In the event of poor visibility on US, additional modalities are required. During the FGD, there was consensus that image fusion could facilitate the localization and targeting of those lesions. Also, it was stated that the choice of the imaging modality, used for needle placement, depends on a case-by-case basis.

### Navigational Tools

Four of the respondents integrated advanced navigational systems from seven different vendors into their clinical practice, while two respondents were exclusively using them within research projects. Considering the static nature of the CT-images that are currently being used for needle navigation, the importance of respiratory control and potential inaccuracies that arise from alternating liver morphology were once again highlighted during the FGD. There was a widespread agreement that confirming needle position and post-ablation margins with contrast-enhanced (cone bean) CT are crucial for optimization of outcomes.

### Treatment Confirmation

To assess technical success, six of the respondents primarily used side-by-side visual (‘eye-balling’) qualitative assessment. Two respondents used visual three-dimensional assessment of ablative margins via pre- and post-ablation CT coregistration. The remaining two respondents employed quantitative assessment of the ablative margins with coregistration. The FGD considered quantitative margin analyses as a method with great potential, but further research is required before it can be widely implemented for clinical decision-making.

## Discussion

Many developments and improvements in tools and software have become available in the field of TA of liver malignancies. Several studies have reported improved outcomes with their implementation [8, 9, 12].

Current clinical practice, however, demonstrates that the enhanced availability of these advancements seems to result in unwanted increased workflow variation. This is mirrored in the variability in treatment strategies observed among the participants in the FGD. A more standardized approach of TA is therefore essential to reduce operator-dependency and guarantee access to high-standard care for patients worldwide. Navigational systems, robotics and planning and confirmation software may help to standardize essential elements of the procedure such as needle placement and assessment of technical success. However, the adoption of such technology is currently heterogeneous and may paradoxically lead to more practice variation.

Different imaging modalities can be used for needle placement and the FGD brought forward that the modality of choice is best determined on a case-by-case basis, while none of the techniques is superior over the others in all cases. Irrespective of the modality used, confirmation of needle position as well as immediate post-ablation confirmation of adequate ablative margins were deemed critical. Three-dimensional planning can be beneficial when requiring the placement or repositioning of multiple probes [13].

The majority of participants used eye-balling comparison for treatment evaluation. This is remarkable as visual qualitative assessment has poor intra and interobserver variability and may lead to misjudgments in up to 44% of cases [9, 12]. Various quantification software packages are available to (semi-)automatically evaluate treatment margin [8]; yet, the results of the FGD indicate that the methodology is still not widely adopted in clinical practice and needs validation in clinical trials [14–16]. Besides, additional research on ablative tissue shrinkage is needed, as this may lead to underestimation of ablation margins.

This study has several limitations. Firstly, selection bias due to the purposive sampling with imbalanced nationality and expertise among the participants may limit the global applicability. Additionally, the incomplete questionnaire response rate and small number of participants may compromise the data robustness.

## Conclusion

Current diversity in workflow and outcomes of thermal ablation of malignant liver tumors highlights the necessity for standardization. While advancements in tools and software hold the potential to homogenize outcome measurement and minimize operator-dependent variability, the enhanced availability also contributes to increased workflow variation.

## Supplementary Information

Below is the link to the electronic supplementary material.

Supplementary file1 (DOCX 26 KB)
