# Supplementary material for: The Paradox of Modern Technology in Standardizing Thermal Liver Ablation: Fostering Uniformity or Diversity?
Source: Cardiovasc Intervent Radiol. 2024 Sep 3;47(10):1402–6. doi: 10.1007/s00270-024-03846-2 (PMC11486791; doi:10.1007/s00270-024-03846-2)
Supplement: Supplementary file 1 — Supplementary file1 (DOCX 26 KB) [file 270_2024_3846_MOESM1_ESM.docx]

**Appendix A, Table 1.** **Results from the cross-sectional survey *.**

|  | | **Question** | **Answer** |
| --- | --- | --- | --- |
| 1. |  | How many liver ablations do you perform on weekly basis? | Average: 3.056; Median 3;  SD 1.102;  IQR 0.5 |
| 2. |  | What is the ratio primary versus secondary malignant liver tumors that you ablate? | Average: 0.55; Median 0.5; SD 0.25, IQR 0.4 |
| 3. |  | Do you think international standardization of malignant liver tumor ablation is important? |  |
|  |  | a) yes | 90% (n = 9) |
|  |  | b) no | 10% (n = 1) |
| 4. |  | Do you think international standardization of malignant liver tumor ablation is reasonably possible? |  |
|  |  | a) yes | 100% (n = 10) |
|  |  | b) no | 0% (n = 0) |
| 5. | 1. | Do you use computer-assisted treatment planning for malignant liver tumor ablation |  |
|  |  | a. Yes | 50% (n = 5) |
|  |  | b. Only in a research setting | 10% (n = 1) |
|  |  | c. No | 40% (n = 4) |
|  | 2. | If question 5.1. answered with ‘yes’, which tool do you use? |  |
|  |  | a. Fusion imaging (US/CT) & Electromagnetic navigation | 20% (n = 1) |
|  |  | b. (CBCT) XperGuide as well as tumor and ablation planning | 20% (n = 1) |
|  |  | c. Plan with MPR following CT hepatic arteriography | 20% (n = 1) |
|  |  | d. 3D Planning & 3D positioning | 20% (n = 1) |
|  |  | e. Neuronavigation with aiming device | 20% (n = 1) |
|  | 3. | In what percentage of the cases? |  |
|  |  | a) All cases (100%) | 80% (n = 4) |
|  |  | b) 20% | 20% (n = 1) |
| 6. | 1. | Which primary imaging modality do you use for needle placement in the majority of your liver ablation procedures? (e.g. US/(CB)CT with catheter in the hepatic artery/fusion imaging/navigation software/other) |  |
|  |  | 1. Ultrasonography | 40% (n = 4) |
|  |  | 2. CT | 20% (n = 2) |
|  |  | 3. (CB)CT with catheter in hepatic artery | 20% (n = 2) |
|  |  | 4. CBCT (with fusion of pre-intervention imaging) | 10% (n = 1) |
|  |  | 5. Fusion imaging CT/US | 10% (n = 1) |
| 7. | 1. | Do you use any needle placement tool (needle holders such as SeeStar, Micromate or laser(s)) for malignant liver tumor ablation |  |
|  |  | a. Yes | 20% (n = 2) |
|  |  | b. Only in a research setting | 0% (n = 0) |
|  |  | c. No | 80% (n = 8) |
|  | 2. | If 7.1. answered with ‘yes’: in what percentage of the cases? ** |  |
|  |  | a. 100% | 100% (n = 2) |
|  | 3. | If 7.1. answered with ‘no’: why don’t you make use of it |  |
|  |  | 1. Preference for free hand | 37.5% (n = 3) |
|  |  | 2. Needle placement tools are unavailable in my institution | 12.5% (n = 1) |
|  |  | 3. Needle placement tools are not always available | 12.5% (n = 1) |
|  |  | 4. I may consider using needle placement tools in the future | 37.5% (n = 3) |
| 8. | 1. | Which ablation solution do you use in the majority of the cases for malignant liver tumor ablation? |  |
|  |  | a. RFA Single needle | 0% (n = 0) |
|  |  | b. RFA Multiple needles | 10% (n = 1) |
|  |  | c. MWA Single needle | 70% (n = 7) |
|  |  | d. MWA Multiple needles | 10% (n = 1) |
|  |  | e. Other | 10% (n = 1) |
| 9. | 1. | Do you use multimodality image fusion during live guidance in malignant liver tumor ablation? (e.g. US/CT-MRI) |  |
|  |  | a. Yes | 70% (n = 7) |
|  |  | b. No | 30% (n = 3) |
|  | 2. | If question 9.1. answered with ‘no’, please provide rationale** |  |
|  |  | a. Not reliable | 33% (n = 1) |
|  |  | b. Not available | 33% (n = 1) |
|  |  | c. Too much effort | 33% (n = 1) |
|  | 3. | If question 9.1. answered with ‘yes’, which imaging modalities are fused? |  |
|  |  | a. CT, MRI, US | 43% (n = 3) |
|  |  | b. CT, MRI, US, PET, SPECT | 14% (n = 1) |
|  |  | c. US, CT | 43% (n = 3) |
|  | 4. | If question 9.1. answered with ‘yes’, in what percentage of the cases? (approximately)** |  |
|  |  | a. >85% | 28.6% (n = 2) |
|  |  | b. 30% | 28.6% (n = 2) |
|  |  | c. 15% | 14.3% (n = 1) |
|  |  | d. 5% | 14.3% (n = 1) |
|  |  | e. 2% | 14.3% (n = 1) |
| 10 | 1. | Do you use any navigation guidance tool/device (e.g. PercuNav, Cascination etc.) for targeting and subsequent thermal liver tumor ablation. |  |
|  |  | a. Yes | 40% (n = 4) |
|  |  | b. Only in a research setting | 20% (n = 2) |
|  |  | c. No | 40% (n = 4) |
|  | 2. | If question 10.1. answered with ‘no’, please provide rationale ** |  |
|  |  | a. Guidance tools have no added benefit in my workflow | 25% (n = 1) |
|  |  | b. Guidance tools are not available in my institution | 25% (n = 1) |
|  |  | c. The costs of guidance tools are too high | 25% (n = 1) |
|  |  | d. Guidance tools are inconvenient | 25% (n = 1) |
|  | 3. | If question 10.1. answered with ‘yes’ or ‘only in research setting’, which tool do you use? (multiple answers may be provided) |  |
|  |  | a. IMACTIS^®^ CT-navigation ^TM^ & ESAOTE (experimental) | 20% (n = 1) |
|  |  | b. IMACTIS^®^ CT-navigation ^TM^ | 40% (n = 2) |
|  |  | c. StealthStation ^TM^ S8 Medtronic | 20% (n = 1) |
|  |  | d. Not specified | 40% (n = 2) |
| 11. |  | What is your opinion on robotic needle placement for thermal liver tumor ablation |  |
|  |  | a) I believe fully automatic robotic needle placement is the future for thermal ablation. | 10% (n = 1) |
|  |  | b) Robotic needle placement is only useful as a needle holding and guidance device | 30% (n = 3) |
|  |  | c) Robotic needle placement may be helpful in challenging cases | 20% (n = 2) |
|  |  | d) Robotic needle placement does not align with my preferences | 20% (n = 2) |
|  |  | e) Other | 20% (n = 2) |
| 12. |  | How do you assess technical success of your thermal liver tumor ablation? |  |
|  |  | a. By visual assessment using side-by-side comparison of pre- and post-ablation margins | 60% (n = 6) |
|  |  | b. By visual assessment using co-registration of the pre- and post-ablation images (fusion imaging) | 20% (n = 2) |
|  |  | c. Quantitative margin analysis using image co-registration of pre- and post-ablation images | 20% (n = 2) |
| 13. |  | What is your opinion on software used for quantitative margin analysis? ** |  |
|  |  | a. Software mediated quantitative margin analysis is unnecessary if manual fusion software is sufficient | 10% (n = 1) |
|  |  | b. Software mediated quantitative margin analysis under general anesthesia (so no motion) is ready for use | 20% (n = 2) |
|  |  | c. Software mediated quantitative margin analysis is very useful in clinical practice | 10% (n = 1) |
|  |  | d. Software mediated quantitative margin analysis is necessary | 50% (n = 5) |
|  |  | e. Use of software mediated quantitative margin analysis is advisable | 10% (n = 1) |
| 14. |  | What do you think about augmented reality (HoloLens or other glasses) for needle guidance? ** |  |
|  |  | a. In my opinion augmented reality is of limited value | 30% (n = 3) |
|  |  | b. Only for educational/training purposes | 20% (n = 2) |
|  |  | c. Interesting, it holds potential for the future | 30% (n = 3) |
|  |  | e. No opinion | 20% (n = 2) |
| 15. |  | Which technological developments do you find most promising to optimize liver ablation? (possibility to provide multiple answers) ** |  |
|  |  | a. Larger, spherical ablations with a single needle | 8.3% (n = 1) |
|  |  | b. Short hospitalizations with sequential treatments | 8.3% (n = 1) |
|  |  | c. Stereotactic approach, including perioperative image fusion. | 8.3% (n = 1) |
|  |  | d. Hybrid (CT) solutions with catheter in hepatic artery/superior mesenteric artery | 16.7% (n = 2) |
|  |  | e. Robotic needle guidance as current practice | 16.7% (n = 2) |
|  |  | f. Computer-assisted planning and treatment evaluation | 8.3% (n = 1) |
|  |  | g. Margin assessment tools | 25% (n = 3) |
|  |  | h. Introduction of (trackable) fiducials for pre-, during- and post treatment evaluation | 8.3% (n = 1) |

**_Table 1 Results from the cross sectional survey._** _SD, standard deviation; IQR, interquartile range; US, ultrasonography; CT, computed tomography; CBCT, cone beam computer tomography; MPR, multiple probe repositioning; 3D, three-dimensional; RFA, radiofrequency ablation; MWA, microwave ablation; MRI, magnetic resonance imaging; PET, positron emission tomography; SPECT, single-photon emission computed tomography._
_* This cross sectional survey was completed by 10 respondents (constituting 77% of the participants of the FGD)._**_**_** _Open-ended question. The answer was provided through a textbox._
